# Mild-intensity physical activity prevents cardiac and osseous iron deposition without affecting bone mechanical property or porosity in thalassemic mice

**DOI:** 10.1038/s41598-022-09997-x

**Published:** 2022-04-08

**Authors:** Narattaphol Charoenphandhu, Supagarn Sooksawanwit, Ratchaneevan Aeimlapa, Natchayaporn Thonapan, Pornpailin Upanan, Punyanuch Adulyaritthikul, Saowalak Krungchanuchat, Nattapon Panupinthu, Jarinthorn Teerapornpuntakit, Catleya Rojviriya, Kornkamon Lertsuwan, Saovaros Svasti, Kannikar Wongdee

**Affiliations:** 1 Department of Physiology, Faculty of Science, Mahidol University, Bangkok, 10400 Thailand; 2 Center of Calcium and Bone Research (COCAB), Faculty of Science, Mahidol University, Bangkok, 10400 Thailand; 3 Institute of Molecular Biosciences, Mahidol University, Nakhon Pathom, 73170 Thailand; 4 The Academy of Science, The Royal Society of Thailand, Bangkok, 10300 Thailand; 5 Molecular Medicine Graduate Program, Faculty of Science, Mahidol University, Bangkok, 10400 Thailand; 6 Faculty of Allied Health Sciences, Burapha University, Long-Hard Bangsaen Road, Chonburi, 20131 Thailand; 7 Department of Physiology, Faculty of Medical Science, Naresuan University, Phitsanulok, 65000 Thailand; 8 Synchrotron Light Research Institute (Public Organization), Nakhon Ratchasima, 30000 Thailand; 9 Department of Biochemistry, Faculty of Science, Mahidol University, Bangkok, 10400 Thailand; 10 Thalassemia Research Center, Institute of Molecular Biosciences, Mahidol University, Nakhon Pathom, 73170 Thailand

**Keywords:** Physiology, Diseases

## Abstract

Thalassemia causes anemia, ineffective erythropoiesis, bone loss and iron accumulation in several tissues, e.g., liver, bone and heart, the last of which leads to lethal cardiomyopathy and arrhythmia. Although exercise reportedly improves bone density in thalassemic mice, exercise performance is compromised and might pose risk of cardiovascular accident in thalassemic patients. Therefore, we sought to explore whether mild-intensity physical activity (MPA) with 30–50% of maximal oxygen consumption was sufficient to benefit the heart and bone. Herein, male hemizygous β-globin knockout (BKO) mice and wild-type littermates were subjected to voluntary wheel running 1 h/day, 5 days/week for 3 months (MPA group) or kept sedentary (SDN; control). As determined by atomic absorption spectroscopy, BKO-MPA mice had less iron accumulation in heart and bone tissues compared with BKO-SDN mice. Meanwhile, the circulating level of fibroblast growth factor-23—a factor known to reduce serum iron and intestinal calcium absorption—was increased early in young BKO-MPA mice. Nevertheless, MPA did not affect duodenal calcium transport or body calcium retention. Although MPA restored the aberrant bone calcium-phosphorus ratio to normal range, it did not change vertebral calcium content or femoral mechanical properties. Microstructural porosity in tibia of BKO-MPA mice remained unaltered as determined by synchrotron radiation X-ray tomographic microscopy. In conclusion, MPA prevents cardiac and bone iron accumulation, which is beneficial to thalassemic patients with limited physical fitness or deteriorated cardiac performance. However, in contrast to moderate-intensity exercise, MPA does not improve bone mechanical properties or reduce bone porosity.

## Introduction

β-Thalassemia is an inherited anemic disease caused by a decrease in or absence of β-globin production, which leads to several adverse consequences, e.g., intestinal iron hyperabsorption, abnormal iron deposition, hepatosplenomegaly, osteopenia and dysregulation of calcium and vitamin D metabolism^1,2^. Since iron is a toxic metal capable of inducing cellular oxidative stress, excess iron accumulation largely contributes to the dysregulation of bone and calcium metabolism and cardiac function in thalassemia^3–5^. We previously reported that an increase in intestinal iron absorption and impaired vitamin D metabolism decreased the intestinal calcium uptake in thalassemic mice^1,6^. Although the reduction in calcium absorption is often explained by thalassemia-associated hypoparathyroidism, low vitamin D production and interference from iron hyperabsorption^1,7,8^, it could also be due to an increase in fibroblast growth factor (FGF)-23. As a bone-derived hormone, FGF-23 functions in mineral regulation, e.g., by enhancing renal phosphate excretion, preventing intestinal calcium absorption, and lowering serum iron^9–11^. Meanwhile, the high rates of intestinal iron absorption, blood transfusion and a massive destruction of senescent red blood cells by the reticuloendothelial macrophages markedly increase the plasma level of non-transferrin-bound iron, which becomes deposited in cells, such as cardiomyocytes, hepatocytes and bone cells^12,13^.

Several lines of evidence have shown a high prevalence of osteopenia in β-thalassemic patients and animal models of the disease^14–17^. Our previous study in β-thalassemic mice showed growth retardation and conspicuously low bone mass^16,18^. Further assessment of bone microstructure and bone cell functions in thalassemic mice by bone histomorphometry revealed decreases in trabecular bone volume, trabecular number and thickness, which resulted from suppression of osteoblast-mediated bone formation and activation of osteoclast-mediated bone resorption^16,18^.

Regular exercise and physical activities are known to have beneficial effects on body systems including iron and bone metabolism^19–21^. Muscle contraction during physical activities provide mechanical stimulation for bone tissue, and in so doing, activates bone cells and consequently enhances bone formation via cytokine release and direct cell-to-cell communication^22^. In addition, muscle contraction also induces the release of myokines, which help improve bone metabolism^23^. In thalassemic animals, endurance exercise was found to improve bone microstructure by enhancing bone formation and suppressing bone resorption^16,24,25^. However, in the real situation, low physical fitness in thalassemic patients is the main hindrance in the use of regular exercises, particularly high-impact exercise regimen. There were reported that thalassemic patients exhibited a marked reduction in exercise performance due to anemia-related fatigue^26,27^. It was not known whether mild physical activity (MPA) such as walking or running with < 50% of maximal oxygen consumption, which should be well tolerated by most thalassemic patients, might provide sufficient benefit to both heart and bone. Thus, MPA may be a recommended choice for promoting health and alleviating iron deposition in cardiomyocytes in thalassemic patients.

Therefore, the present study aimed to investigate the effects of MPA on tissue iron accumulation, and changes in bone structure and mechanical strength in an animal model of thalassemia. The hemizygous β-globin knockout (BKO) thalassemic mice used in the present study exhibited thalassemia intermedia featuring congenital microcytic hypochromic anemia with anisopoikilocytosis, consistent with the phenotype of β-thalassemic patients^28^. Because BKO mice had anemia that made them easily fatigue during physical exertion, we used mild-intensity physical activity in the present study to let them gain some benefits from MPA. Although we are aware that, under normal conditions, moderate-to-high intensity exercise is required to produce conspicuous outcomes on bone and cardiovascular system, we expected to see certain benefits of MPA that helped reduce bone and cardiac iron accumulation in BKO mice.

## Materials and methods

### Animals

Four-week-old male hemizygous β-globin knockout thalassemic mice (BKO) and C57BL/6 wild-type (WT) littermates were obtained from the Thalassemia Research Center, Institute of Molecular Biosciences, Mahidol University, Thailand. Our BKO mouse strain was *Hbb*^th-3^/*Hbb*^+^, also known as *Hbb-b1*^tm1Unc^ *Hbb-b2*^tm1Unc^ (β^major^- and β^minor^-globin-targeted deletion knockout). In other words, they were hemizygous mice that one of two β-globin copies was deleted, similar to Jackson Laboratory mouse strain #002683. Since the homozygous BKO genotype is lethal, the hemizygous mouse strain was used in this study. The animals were housed in polystyrene cages (2–4 animals per cage) with relative humidity of 50–60% and room temperature of 22 ± 1 °C in an animal husbandry unit accredited by Association for Assessment and Accreditation of Laboratory Animal Care International (AAALAC). They were fed standard laboratory chow (cat. no. 082G; Perfect Companion Group Co., Ltd., Bangkok, Thailand) and reverse osmosis water ad libitum. Venous blood from the tail vein was collected from each mouse for blood smear analysis (Wright-Giemsa staining) and automated complete blood count (CBC) to confirm the characteristics of thalassemic anemia. The presence of bone microstructural defect in BKO vs. WT mice was confirmed by bone histomorphometry. At the end of experiments, all animals were euthanized by intraperitoneal injection of 5 mg/kg xylazine (X-lazine; L.B.S. Laboratory Ltd., Part, Bangkok, Thailand) and 40 mg/kg tiletamine/zolazepam (Zoletil™ 100; Virbac Laboratories, Carros, France). The animal experiment protocol has been approved by the Institutional Animal Care and Use Committee of the Faculty of Science, Mahidol University. All experiments were performed in accordance with relevant regulations and the ARRIVE (Animal Research: Reporting on In Vivo Experiments) guideline.

### Experimental design

After a week of acclimatization, 5-week-old WT and BKO mice were randomly divided into MPA and sedentary (SDN) groups. WT littermates were used as controls. The animals underwent MPA training schedule for 1, 2 or 3 months. A researcher was assigned to closely inspect and monitor all animals during the entire training period. Body weight and food intake were recorded, and feces and urine were collected for calcium balance study. Thereafter, animals were euthanized on the designated day. Blood was collected for hematological analysis to confirm the characteristics of thalassemia and determination of FGF-23, osteokine and myokine levels. After euthanasia, gastrocnemius muscle, liver, spleen and heart were collected and weighed. Heart and spleen tissues were processed histologically for Prussian blue staining to visualize iron accumulation. The images were captured from areas that represented the whole slide. Liver, spleen and heart tissues were further examined for tissue iron content, and L4-5 lumbar vertebrae were determined for calcium, phosphate, and iron contents. Initially, a sign of cardiac iron accumulation was screened by using chromogenic method, as reported in Table 1. After we found positive data of iron overload, more sensitive technique, i.e., flame atomic absorption spectrometry (FAAS), was used to measure iron contents. In some experiments, the duodenal tissues were collected for measurement of the calcium absorption rate and epithelial electrical parameters by Ussing chamber technique. Femora were collected for assessment of bone mechanical properties by computer-assisted three-point bending test. Additionally, tibiae were assessed for bone porosity by Synchrotron radiation X-ray tomographic microscopy (SRXTM).

**Table 1:** Blood counts of 2- and 5-month-old male BKO and WT mice.

Parameters	2-month-old mice		5-month-old mice	
	WT (n = 4)	BKO (n = 3)	WT (n = 4)	BKO (n = 3)
**Blood parameters**				
WBC (× 10^3^ cells/µL)	3.80 ± 0.62	6.93 ± 1.89	2.50 ± 0.36	3.59 ± 1.09
RBC (× 10^6^ cells/µL)	6.35 ± 0.21	5.79 ± 0.20	7.50 ± 0.20	6.92 ± 0.10*
Hb (g/dL)	8.20 ± 0.29	5.07 ± 0.24*	10.30 ± 0.29	5.87 ± 0.33*
Hct (%)	31.75 ± 0.85	25.27 ± 1.75*	37.00 ± 0.88	27.40 ± 0.60*
MCV (fL)	51.13 ± 0.78	43.63 ± 1.79*	49.33 ± 0.45	39.63 ± 0.69*
MCH (pg)	13.95 ± 1.00	8.67 ± 0.13*	13.75 ± 0.22	8.87 ± 0.12*
MCHC (g/dL)	25.95 ± 0.70	19.97 ± 0.58*	27.83 ± 0.25	22.47 ± 0.33*
RDW (%)	13.90 ± 0.14	37.17 ± 1.01*	14.00 ± 0.12	37.57 ± 1.56*
Plt (× 10^3^ cells/µL)	735.5 ± 115	1142.0 ± 192	446.0 ± 110	687.3 ± 42.73
MPV (fL)	6.18 ± 0.23	7.10 ± 0.63	6.23 ± 0.05	6.17 ± 0.19
PDW (%)	46.93 ± 2.61	53.53 ± 1.83	37.95 ± 1.27	50.60 ± 1.91*
**Tissue iron content (mg Fe/g dry weight)**				
Splenic Fe	0.23 ± 0.10	1.99 ± 0.41*	1.71 ± 0.20	4.66 ± 0.68*
Hepatic Fe	0.04 ± 0.01	0.99 ± 0.11*	0.15 ± 0.01	0.59 ± 0.08*
Cardiac Fe	0.12 ± 0.02	0.16 ± 0.01	0.12 ± 0.00	0.15 ± 0.01*

Values are mean ± SE.

*WT* wild-type, *BKO* hemizygous β-globin knockout thalassemia, *WBC* white blood cell count, *RBC* red blood cell count, *Hb* hemoglobin concentration, *Hct* hematocrit, *MCV* mean corpuscular volume, *MCH* mean corpuscular hemoglobin, *MCHC* mean corpuscular hemoglobin concentration, *RDW* red cell distribution width (variation in red blood cell size or volume), *Plt* Platelet count, *MPV* mean platelet volume, *PDW* platelet distribution width.

*P < 0.05 BKO vs. age-matched WT mice. The tissue iron contents were analyzed by chromogenic method.

### Mild-intensity physical activity (MPA) training

In the MPA group, mice were subjected to walk or run voluntarily in running wheels (model 80800A, Lafayette Instrument, Lafayette, IN, USA) to increase their physical activity. The MPA schedule was 5 days/week for a period of 1, 2 or 3 months. The duration and speed were adjusted so that BKO mice were able to perform MPA training without signs of exhaustion or severe fatigue. Initially, MPA mice were trained for 30–50 min per day at a speed of less than 4 m/min. In the second week, the running duration was increased to 60 min/day. The present MPA protocol corresponded to 30–50% of maximal oxygen consumption. Running speed was continuously maintained at 4–6 m/min until the end of the experiment. For SDN group, animals remained sedentary in their cages throughout the study period. Body weights of all animals were recorded weekly.

### Measurement of tissue iron, calcium and phosphate contents

In the beginning, 2- and 5-month-old male BKO mice were checked for the presence of anemia and tissue iron accumulation by using CBC and iron chromogenic assay, respectively. Regarding the iron chromogenic assay, the liver, spleen and heart tissues were subjected to freeze drying, followed by incubation in 3 M hydrochloric acid and 10% trichloroacetic acid for 20 h at 65 °C. After centrifugation, the supernatants were added into a 96-well plate filled with chromogenic solution (0.1% bathophenanthroline disulfonic acid disodium salt hydrate, 1% thioglycolic acid and 3.39 M sodium acetate trihydrate) and read by a spectrophotometer at 535 nm.

In the MPA study, a highly sensitive technique, i.e., atomic absorption spectroscopy, was used. The liver, spleen, heart and L4-5 lumbar vertebrae were removed, weighed and dried at 80 °C for 3 days to obtain the final dry weight. Bone specimens were milled in liquid nitrogen. Bone and tissue powder were digested in a mixture of 30% hydrogen peroxide (H_2_O_2_) and 65% vol/vol nitric acid (HNO_3_) in Ethos UP MAXI-44 microwave digester (Milestone, Shelton, CT, USA). Calcium and iron contents were measured by flame atomic absorption spectroscopy (FAAS; PinAAcle 900 T, PerkinElmer), and phosphorus was analyzed by graphite furnace atomic absorption spectroscopy (GFAAS; Agilent 240Z AA, USA) with 2% vol/vol HNO_3_ as a blank solution.

### Measurement of serum FGF-23 and other humoral factors

The level of intact FGF-23 was determined by commercial ELISA kit (cat. no. 60-6800; Immutopics, Quidel Corporation, San Diego, CA, USA). Serum myokines/osteokines [i.e., myostatin, osteocrin, osteonectin, fibroblast growth factor-21 (FGF-21), follistatin-like protein 1 (FSTL-1) and irisin, all of which are secreted by muscle and/or bone] were determined by milliplex map mouse myokine magnetic bead panel (cat. no. MMYOMAG-74 K; Millipore, MA, USA). All procedures were performed according to the manufacturers’ instructions.

### Measurement of the transepithelial calcium flux by radiotracer

Transepithelial calcium flux was determined by the Ussing chamber technique as previously described^1,29^. In brief, duodenum was cut longitudinally and rinsed with isotonic bathing solution that contained (in mM) 118 NaCl, 4.7 KCl, 1.1 MgSO_4_, 1.25 CaCl_2_, 23 NaHCO_3_, 12 d-glucose, and 2 mannitol (Sigma, St. Louis, MO, USA). The duodenum was then mounted between two hemichambers and incubated for 10 min prior to incubating with bathing solution containing radioactive tracer. During calcium flux measurement, the apical side of duodenal tissue was exposed to the bathing solution containing ^45^Ca (initial amount of 0.451 Ci/mL, final specific activity of 90 mCi/mol; cat. no. NEZ013, PerkinElmer, Boston, MA, USA), whereas the basolateral side was exposed to radiotracer-free bathing solution. Two samples from the hot side (H; apical solution) were collected at 10 and 20 min, other five samples were collected from the cold side (C; basolateral solution) every 10 min until the end of the experiment. Unidirectional calcium flux (*J*_H→C_; nmol h^−1^ cm^−2^) from the hot side to the cold side was calculated using Eqs. (1) and (2):

1 $$J_{{{\text{H}} \to {\text{C}}}} = R_{{{\text{H}} \to {\text{C}}}} /\left( {S_{{\text{H}}} \times A} \right),$$

2 $$S_{{\text{H}}} = C_{{\text{H}}} /C_{{\text{T}}} ,$$

where *R*_H→C_ is the rate of ^45^Ca appearance in the cold side (cpm h^−1^); *S*_H_, specific activity in the hot side (cpm nmol^−1^); *A*, epithelial surface area (cm^2^); *C*_H_, mean radioactivity in the hot side (cpm); and *C*_T_, total calcium content in the hot side (nmol).

Radioactivity of ^45^Ca (beta radiation) in each sample was quantified by liquid scintillation spectrophotometer (model Tri-Carb 3100 Packard; PerkinElmer Life and Analytical Sciences Shelton, CT, USA). In the absence of the transepithelial calcium gradient, i.e., free-ionized calcium concentration in both apical and basolateral hemichambers was 1.25 mM, the measured calcium flux represented active calcium transport^30^.

### Measurement of epithelial electrical properties in duodenum

Electrical parameters of the duodenal epithelium, i.e., potential difference (PD), short-circuit current (*I*_sc_) and transepithelial resistance (TER) were used to assess changes in electrogenic ion transport and epithelial integrity^1^. PD and *I*_sc_ were measured by two pairs of electrodes made of Ag/AgCl half-cells connecting to the Ussing chamber via salt bridges (3 M KCl in 3% agar). To measure PD, the electrodes were placed near the mounted epithelial sheet and connected to pre-amplifier (model EVC-4000, World Precision Instruments, Sarasota, FL, USA) and PowerLab 4/30 (ADInstruments, Colorado Springs, CO, USA). Another pair of electrodes was placed at the other side as far as possible from the mounted tissues to supply short-circuit current (*I*_sc_), which equivalent to the total current due to electrogenic ion transport. *I*_sc_ measurement was performed using PowerLab 4/30 connected in series to the EVC-4000 current-generating unit and operated with Chart 5.2.2 for Mac OS X (ADInstruments). TER was calculated from Ohm’s equation.

### Localization of FGF-23 in duodenal epithelium by confocal laser-scanning microscopy

To visualize the distribution of FGF-23 and confirm its existence in the intestinal epithelium, the duodenal tissues were collected from 24-week-old female rats, and fixed in 4% formaldehyde solution at 4 °C for 3 h. Fixed tissue was washed with phosphate-buffered saline (PBS), immersed in 20% sucrose in PBS at 4 °C and embedded in optimal cutting temperature (OCT) compound (Sakura Finetek, Netherlands) and finally cut into 8-µm sections by a cryostat (model CM1800, Leica microsystems, IL, USA). Non-specific binding was blocked by 2-h incubation with blocking solution (4% bovine serum albumin, 5% normal goat serum and 0.1% Tween-20 in PBS), followed by overnight incubation with 1:100 rabbit anti-FGF-23 primary antibody (cat. no. PA5-77259; Thermo Fisher Scientific, Waltham, MA, USA; RRID: AB_2720986). This anti-FGF-23 antibody was demonstrated to produce a single band in western blot analysis for mouse and rat brain, which has been known to express FGF-23 protein^31^. After being washed, the sections were incubated at room temperature for 1 h with 1:500 goat anti-rabbit Dylight-594-conjugated secondary antibody (DI-1594; Vector Laboratories, Burlin-game, CA, USA), and 1:1000 phalloidin-conjugated iFluor 448 (ab176753; Abcam, Cambridge, MA, USA). Nuclei were stained with 4′,6-diamidino-2-phenylindole in antifade mountant (S36964; Thermo Fisher Scientific). Images were captured using a confocal laser-scanning microscope (model Zeiss LSM800; Carl Zeiss AG, Germany) and processed with Zeiss ZEN Blue software. Bone specimens (femoral cortical envelope) containing osteocytes—i.e., FGF-23-positive cells—were used to validate FGF-23 antibody. In this validating experiment, fluorescent signals in bone sections were visualized by a multiphoton microscope (model SP8 DIVE; Leica Microsystems, Germany).

### Calcium balance study

Calcium balance study was performed in the last week of the 3-month MPA program to determine total body calcium retention and excretion. Mice were placed individually in metabolic cages for 48 h to monitor food intake, urine and fecal excretion. Fecal pellets were collected, weighed and dried in an oven at 80 °C and ashed at 800 °C overnight in a muffle furnace (model 48000; Thermolyne, Dubuque, IA, USA). Dry samples were then digested in Ethos UP MAXI-44 microwave digester (Milestone, CT, USA). Calcium in urine and feces was measured by PinAAcle 900 T FAAS (PerkinElmer). Fecal calcium excretion (F_Ca_; mmol/100 g body weight/day) was calculated from calcium in total ash weight, and urinary calcium excretion (U_Ca_; mmol/100 g body weight/day) was calculated from calcium in total urine volume. Percentage of total calcium excretion and retention was calculated using Eqs. (3) and (4):

3 $$\% {\text{ Ca excretion}} = \left( {{\text{U}}_{{{\text{Ca}}}} + {\text{ F}}_{{{\text{Ca}}}} } \right)/{\text{C}}_{{{\text{Ca}}}} \times {1}00$$

4 $$\% {\text{ Ca retention}} = \left[ {{\text{C}}_{{{\text{Ca}}}} {-}\left( {{\text{U}}_{{{\text{Ca}}}} + {\text{F}}_{{{\text{Ca}}}} } \right)} \right]/{\text{C}}_{{{\text{Ca}}}} \times {1}00$$

where C_Ca_ is dietary calcium intake (mmol/100 g body weight/day).

### Assessment of bone mechanical properties

Three-point bending system (model 5943; Instron, Norwood, MA, USA) was used to measure the flexional stiffness and strength of femur, as described previously^32^. Force (2 N; displacement rate of 0.5 mm/s) was applied onto the mid-diaphysis of the left femur, which was initially placed on two supports 6 mm apart and with the femoral anterior margins facing down toward the actuator. A load–displacement curve of each specimen was constructed by Instron 5900 software. The obtained parameters were maximum load, yield load, ultimate load and stiffness.

### Assessment of bone porosity by synchrotron radiation X-ray tomographic microscopy (SRXTM)

After being collected, each tibial specimen was placed in a sample holder filled with cotton balls soaked in PBS to protect bone tissue from dehydration and dislocation during measurement. SRXTM experiments were performed at the X-ray Tomographic Microscopy beamline (BL1.2 W: XTM) of the Siam Photon Source (SPS), Synchrotron Light Research Institute (SLRI), Nakhon Ratchasima, Thailand. The synchrotron radiation was generated from 2.2-T multipole wiggler (1.2 GeV at 150 mA). The image attainment and parameter setup were modified from the method of Tiyasatkulkovit et al.^33^. All tomography datasets were collected with filtered polychromatic X-ray beam at the mean energy of 10.5 keV. The X-ray projections were obtained from the detection system including YAG-Ce scintillator, objective lens-coupled microscope (OptiquePeter, France) and PCO.edge 5.5 camera (PCO Imaging, Germany). The image pixel size was 3.61 × 3.61 µm^2^. Bone image processing and tomographic reconstruction were carried out by Octopus Reconstruction software (Kohoutovice, Czechia). Finally, 3D images were rendered by Drishti software (Canberra, Australia).

Regarding the porosity measurement, the obtained data were analyzed from reconstructed slices using Octopus Analysis software (Kohoutovice, Czechia). The fraction of bone porosity (% porosity) was calculated from the void volume over the analyzed volume (referred to as the volume of interest; VOI), which was averaged from width, height and length of all bone slices from each bone sample.

### Bone histomorphometry

Bone microstructural defect in 4-month-old male BKO vs. WT mice was confirmed by bone histomorphometry, using OsteoMeasure system (version 4.10; OsteoMetrics, Atlanta, GA, USA) equipped with Eclipse Ni-U light/fluorescent microscope (Nikon), as previously described^16,34^. Trabecular bone microstructural parameters consisted of trabecular bone volume normalized by tissue volume (bone volume fraction, BV/TV; %), trabecular number (Tb.N; mm^−1^), trabecular separation (Tb.Sp; µm) and trabecular thickness (Tb.Th; µm). The parameters related to osteoblast and osteoclast functions included osteoblast surface normalized by bone surface (Ob.S/BS; %), osteoid thickness (O.Th; μm), osteoclast surface (Oc.S/BS; %) and active erosion surface (aES/BS; %).

### Statistical analysis

All data are presented as means ± SE. Sample size calculation was performed by Minitab 16 to ensure minimal use of animals. We used number of red blood cells as a parameter for calculating sample size because this parameter was critical for indicating anemia in BKO mice and is known to have large variation. Unless otherwise specified, two groups of data were compared by Student’s *t*-test, whereas comparisons among multiple groups were performed by two-way analysis of variance (ANOVA) with Bonferroni’s multiple comparisons test using GraphPad Prism 9 (GraphPad Software, San Diego, CA, USA). The level of significance for statistical tests was *P* < 0.05.

## Results

### BKO mice manifested anemia with anisopoikilocytosis, bone loss and iron accumulation in various organs

Prior to the MPA experiments, BKO mice were examined by blood smear (data not shown) and CBC (Table 1) for overt signs of hypochromic microcytic anemia with anisocytosis and poikilocytosis, i.e., decreases in hemoglobin level, hematocrit, mean corpuscular volume, mean corpuscular hemoglobin, mean corpuscular hemoglobin concentration, and percentage of red cell distribution width, as compared to WT mice. At 4 months of age, all male BKO mice were found to fully develop signs of trabecular bone microstructural defects in tibial metaphysis (Supplementary Fig. S1), such as decreases in trabecular bone volume, trabecular number and trabecular thickness, and an increase in trabecular separation. When compared to WT mice, this thalassemic bone loss was associated with greater osteoclast surface and active erosion surface, with no change in osteoblast surface or osteoid thickness (Supplementary Fig. S2). Thus, the present phenotype of BKO mice was consistent with thalassemia intermedia and thalassemia-associated osteopathy as reported previously^16^.

Since iron accumulation in vital organs—especially the heart—posed a high risk for lethal complications, we determined the iron contents in liver, spleen and heart to confirm the presence of aberrant iron deposit (Table 1). The hepatic and splenic iron contents were apparently greater in 2- and 5-month-old BKO mice compared with their WT littermates. A significant increase in cardiac iron content was observed in 5-month-old BKO mice, but not in 2-month-old BKO mice (Table 1).

### MPA reduced iron accumulation in cardiac and osseous tissues

**Figure 1:**
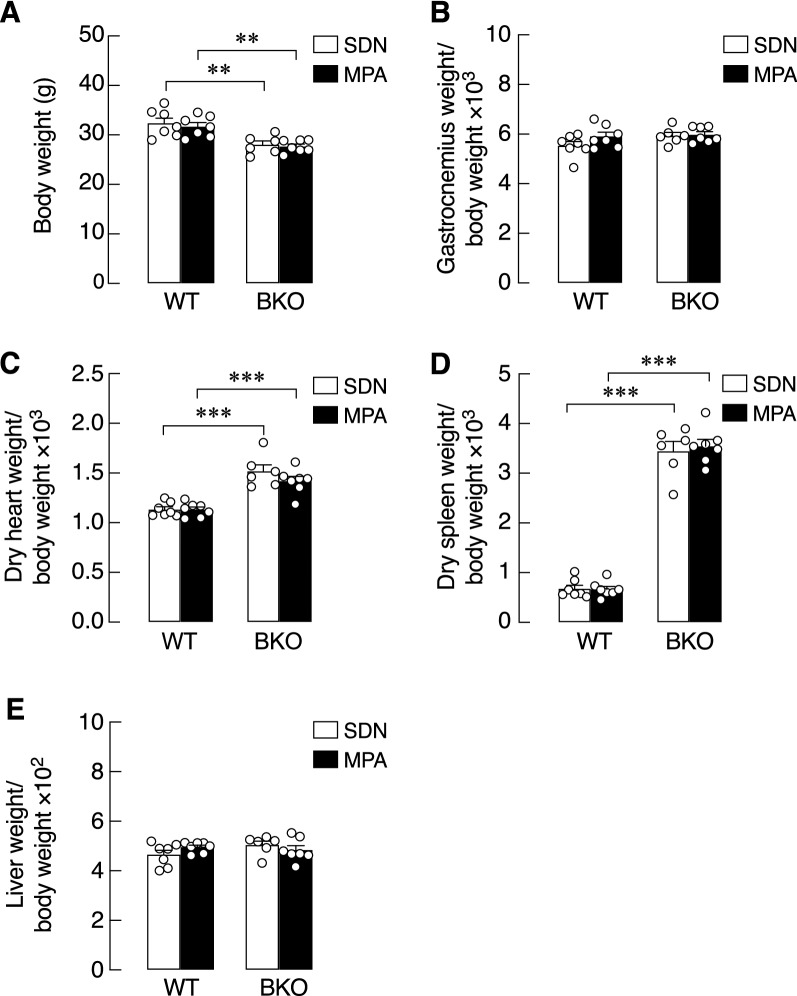
Body weight and organ weights of hemizygous β-globin knockout (BKO) and wild-type (WT) mice after mild-intensity physical activity (MPA) training or sedentary (SDN) period. Body weight (**A**), gastrocnemius weight normalized by body weight (BW) (**B**), dry heart weight normalized by BW (**C**), dry spleen weight normalized by BW (**D**), and wet liver weight normalized by BW (**E**). Values are means ± SE (n = 6–7; **P < 0.01 and ***P < 0.001 compared with corresponding age-matched WT.

In this series of experiments, 5-week-old WT and BKO mice were subjected to either MPA or kept sedentary for 3 months. Body weights and food intake were monitored throughout the experiment. We found that there was no difference in food intake or initial body weights of WT and BKO in both SDN and MPA groups (Table 2). Although the body weights of BKO mice were slightly lower than those of WT mice, the body weight of each group was not affected by 3-month MPA (Table 2 and Fig. 1A), confirming that intensity of the present training protocol was considered as a mild physical activity.

**Table 2:** Initial body weight of WT and BKO mice, body weight gain and food intake of WT and BKO mice after sedentary (SDN) or mild-intensity physical activity (MPA) periods.

Parameters	Experimental groups			
	SDN		MPA	
	WT (n = 7)	BKO (n = 6)	WT (n = 7)	BKO (n = 7)
Initial body weight (g)	15.88 ± 1.52	11.83 ± 2.01	15.67 ± 1.42	13.55 ± 0.87
Body weight gain (g)	13.91 ± 2.11	15.72 ± 2.05	15.67 ± 0.93	15.05 ± 1.07
Food intake (g/day)	3.02 ± 0.14	3.08 ± 0.12	3.22 ± 0.03	3.07 ± 0.07

Data are mean ± SE.

*WT* wild-type, *BKO* hemizygous β-globin knockout thalassemia.

Furthermore, the muscle (gastrocnemius) and internal organ weights of both BKO and WT mice in SDN and MPA groups were determined. As depicted in Fig. 1B–E, the heart and spleen weights of BKO mice were apparently higher, whereas the liver and gastrocnemius weights were similar to those of WT mice. MPA affected neither muscle nor internal organ weights (Fig. 1B–E). The levels of myokines and osteokines are presented in Table 3.

**Table 3:** Serum myokine and osteokine levels of WT and BKO mice after SDN and MPA.

Parameters	Sedentary (SDN)						Mild-intensity physical activity (MPA)					
	1 month		2 months		3 months		1 month		2 months		3 months	
	WT (n = 7)	BKO (n = 3)	WT (n = 7)	BKO (n = 3)	WT (n = 7)	BKO (n = 6)	WT (n = 7)	BKO (n = 6)	WT (n = 7)	BKO (n = 5)	WT (n = 7)	BKO (n = 7)
Myostatin (pg/mL)	1070 ± 120.1	744.1 ± 318.5	1226 ± 140.3	1023 ± 127.5	1550 ± 443.9	1040 ± 91.24	961.6 ± 175.9	1495 ± 84.75^†^	914.3 ± 147.1	809.3 ± 122.4	1378 ± 85.92	1261 ± 58.05
Osteocrin (pg/mL)	130.4 ± 34.31	69.69 ± 18.61	140.6 ± 22.11	96.42 ± 28.91	101.4 ± 18.48	116.1 ± 11.89	107.1 ± 12.29	108.2 ± 22.39	134.4 ± 9.107	59.16 ± 14.08*	119.7 ± 12.50	133.5 ± 20.47
Osteonectin (ng/mL)	61.31 ± 7.32	83.30 ± 8.30	29.92 ± 3.51	43.35 ± 11.69	25.34 ± 3.32	48.74 ± 7.15**	39.45 ± 4.49^#^	64.90 ± 6.63*	28.86 ± 2.57	33.84 ± 7.79	28.38 ± 2.66	36.61 ± 4.06
FGF-21 (pg/mL)	91.96 ± 33.61	51.27 ± 24.61	128.9 ± 76.22	241.0 ± 115.1	22.99 ± 8.43	36.84 ± 18.05	52.80 ± 37.82	35.36 ± 16.30	19.14 ± 7.03	27.78 ± 16.62	17.71 ± 11.19	32.57 ± 12.92
FSTL1 (pg/mL)	3011 ± 427.2	1634 ± 445.0	2925 ± 593.1	638.0 ± 519.7	2638 ± 1144	1478 ± 909.4	4297 ± 1077	3046 ± 2495	2747 ± 890.8	536.1 ± 442.1	3476 ± 742.5	570.4 ± 416.3*
Irisin (pg/mL)	23.19 ± 3.02	26.71 ± 7.16	66.59 ± 24.97	19.02 ± 1.22	49.37 ± 26.69	44.67 ± 7.07	136.3 ± 68.95	31.76 ± 7.16	25.43 ± 4.03	23.14 ± 4.49	239.0 ± 220.0	32.53 ± 6.37

Data are mean ± SE.

*WT* wild-type, *BKO* hemizygous β-globin knockout thalassemia, *FGF-21* fibroblast growth factor-21, *FSTL1* follistatin-like protein 1.

*P < 0.05, **P < 0.01 compared with corresponding WT group.

^#^*P* < 0.05 compared with age- and genotype-matched SDN group.

^†^*P* < 0.05 for the interaction of genotype and activity in similar age group (two-way ANOVA with Bonferroni’s multiple comparisons test).

**Figure 2:**
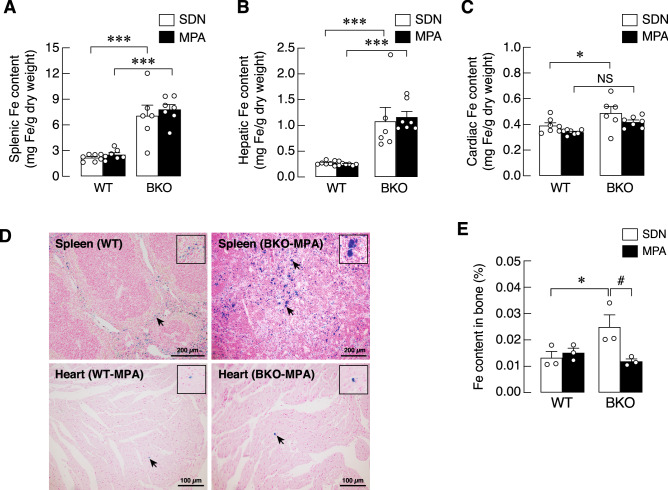
Tissue iron content in β-globin knockout (BKO) and wild-type (WT) mice after mild-intensity physical activity (MPA) training or sedentary (SDN) period. (**A**–**C**) Tissue iron (Fe) content in spleen, liver, and heart were measured by FAAS and normalized by tissue dry weight (n = 6–7). NS, not significant. (**D**) Representative photomicrographs of Prussian blue staining in the spleen and heart tissue obtained from WT and BKO mice (n = 6–7). Iron accumulation stained blue, nuclei stained red. Scale bars are presented in the lower right of each picture. (**E**) Iron content in bone was analyzed by FAAS (n = 3). Values are means ± SE. *P < 0.05, ***P < 0.001 vs. corresponding age-matched WT group. ^#^*P* < 0.05 vs. SDN group.

We further investigated whether MPA was able to alleviate aberrant iron deposition in the hepatic, splenic, cardiac and bone tissues. It was found that MPA did not affect the iron contents in the spleen or liver of WT and BKO animals (Fig. 2A,B). Although the cardiac iron content of BKO-SDN was higher than that of WT-SDN group, the different was absent in BKO-MPA vs. WT-MPA (Fig. 2C; *P* > 0.05). Representative photomicrographs of Prussian blue staining of splenic and cardiac tissues are depicted in Fig. 2D. Interestingly, as compared to WT-SDN mice, the BKO-SDN mice exhibited greater bone iron content, which was completely restored to normal level by MPA (Fig. 2E). Therefore, despite being a mild intensity training, MPA successfully reduced iron accumulation in the cardiac and bone tissues.

### MPA led to the earlier onset of FGF-23 release in BKO mice

**Figure 3:**
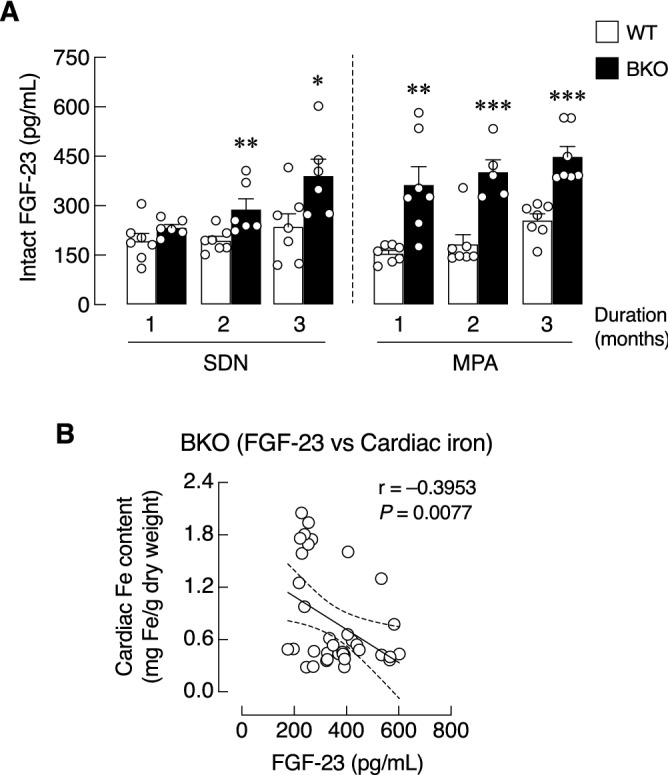
(**A**) Serum level of intact FGF-23 in β-globin knockout (BKO) and wild-type (WT) mice after mild-intensity physical activity (MPA) training for 1, 2, and 3 month(s) compared with corresponding sedentary (SDN) group. Values are means ± SE (n = 5–7; *P < 0.05, **P < 0.01, ***P < 0.001 vs. corresponding age-matched WT group). (**B**) Correlation plot between serum intact FGF-23 and cardiac iron content. Dashed line represents the 95% confidence bands of the best-fit line. r, correlation coefficient. *P*, p-value of the regression.

Since FGF-23 has been reported to be a hypoferremic factor that might be a compensatory protective factor against iron overload^9,35^, we sought to determine the time-dependent changes in the serum intact FGF-23 levels in both SDN and MPA mice (Fig. 3A). The results showed that serum FGF-23 levels of WT mice remained unchanged in both SDN and MPA groups. Strikingly, the onset of serum FGF-23 elevation was observed at 2 months in BKO-SDN group, and at 1 month in BKO-MPA group. In other words, MPA appeared to induce an earlier rise in serum FGF-23 in BKO mice. Furthermore, a correlation study in BKO mice revealed that serum FGF-23 levels were inversely correlated with cardiac iron content (*P* = 0.0077; Fig. 3B), concurring with the hypothesis that FGF-23 was probably a compensatory factor during iron overload.

### MPA did not alter the intestinal calcium absorption in BKO mice

**Figure 4:**
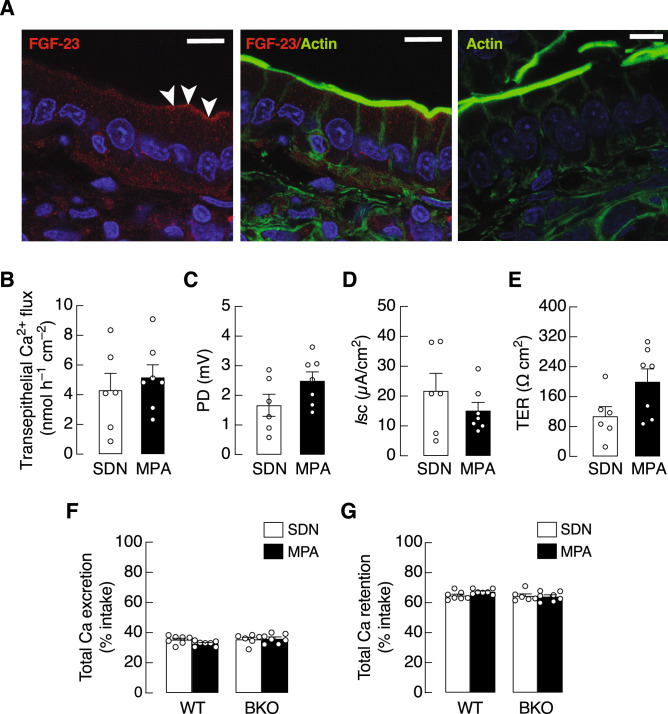
(**A**) Representative photomicrographs of FGF-23 protein expression in duodenal epithelium obtained from confocal laser scanning microscope. Positive red signals of FGF-23 (left and middle panels) are distributed in the cytoplasm especially in the vicinity to the basolateral and apical membrane (arrowhead). Negative control image is presented (right panel). A representative image of actin staining (right panel) is from the same animal (different tissue section), and shows no red signal artifact. Green signal represents F-actin, blue signal represents nucleus (scale bar, 10 µm). (**B**–**E**) Duodenal calcium flux and epithelial electrical parameters, i.e., transepithelial potential difference (PD), short-circuit current (*I*_sc_), and transepithelial resistance (TER) of the duodenal tissues from β-globin knockout (BKO) mice subjected to mild-intensity physical activity (MPA) training or sedentary (SDN) group. Calcium balance study, i.e., total Ca excretion (**F**), and retention (**G**) were determined by metabolic cage study and FAAS. Values are means ± SE (n = 6–7).

Although regular exercise, e.g., forced endurance running, has been known to improve the intestinal calcium absorption^36^, MPA as a mild-intensity physical activity together with the elevated FGF-23 levels were expected to induce different response in BKO mice from that in normal animals. Moreover, as depicted in Fig. 4A, the duodenal epithelial cells of rodents exhibited a strong expression of FGF-23—an inhibitor of calcium absorption in the local intestinal control loop (for review, please see^11^)—as determined by a confocal laser-scanning microscope together with FGF-23 antibody, which was validated and could reveal FGF-23 expression in bone tissue (Supplementary Fig. S3). Thus, the present Ussing chamber study in BKO mice confirmed that MPA together with high circulating FGF-23 levels did not have any significant effect on the duodenal calcium transport or epithelial electrical parameters, i.e., PD, *I*_sc_ and TER (Fig. 4B–E). Consistent with the unaltered transepithelial calcium flux, the calcium balance study showed no change in either total calcium excretion (i.e., urinary and fecal calcium excretion) or total calcium retention after MPA (Fig. 4F,G).

### MPA restored the aberrant bone calcium/phosphorus ratio, but not strength or porosity

**Figure 5:**
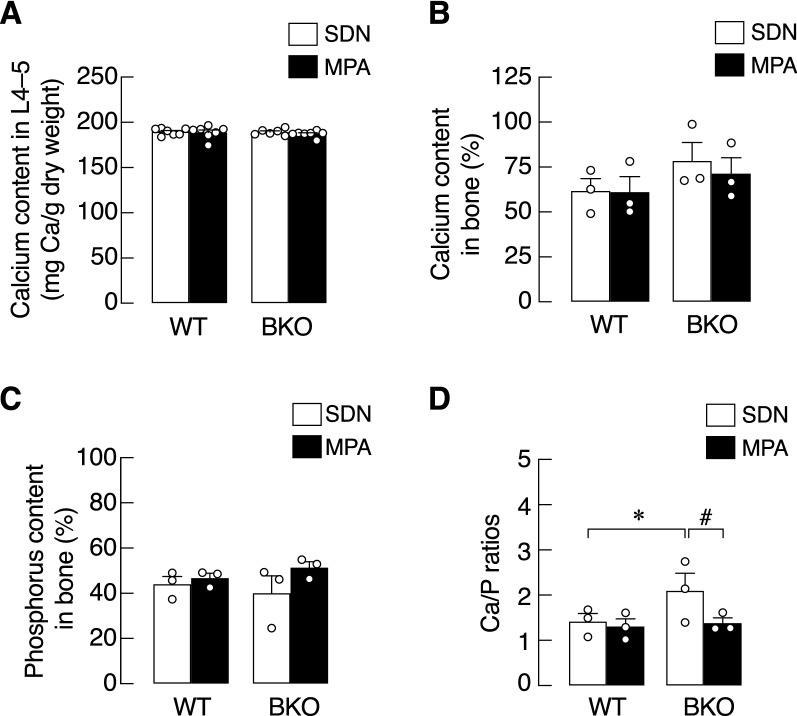
Calcium and phosphorus contents in bone from β-globin knockout (BKO) and wild-type (WT) mice after mild-intensity physical activity (MPA) training or sedentary (SDN) period. (**A**) Calcium content in lumbar vertebrae (L4-5) normalized by tissue dry weight (n = 6–7). (**B**–**D**) Calcium content, phosphorus content, and calcium/phosphate ratio are analyzed by FAAS and GFAAS (n = 3). Values are means ± SE. *P < 0.05 vs. corresponding age-matched WT group. ^#^*P* < 0.05 vs. SDN group.

Since it has been known that toxic metals, including iron^37,38^, are able to interfere with bone calcium and phosphate accretion in bone tissue, we examined calcium and phosphorus contents in the lumbar vertebrae (L4–5) of WT and BKO mice. The total calcium mass as represented by lumbar calcium content per milligram dry weight was similar in both WT and BKO groups, and MPA did not alter this parameter (Fig. 5A). When bone minerals were extracted and analyzed, neither BKO genotype nor MPA showed a significant change in percentage of calcium or phosphorus (Fig. 5B,C). Interestingly, the calcium/phosphorus ratio slightly but significantly increased in the BKO-SDN group, which was completely restored by MPA (Fig. 5D).

**Figure 6:**
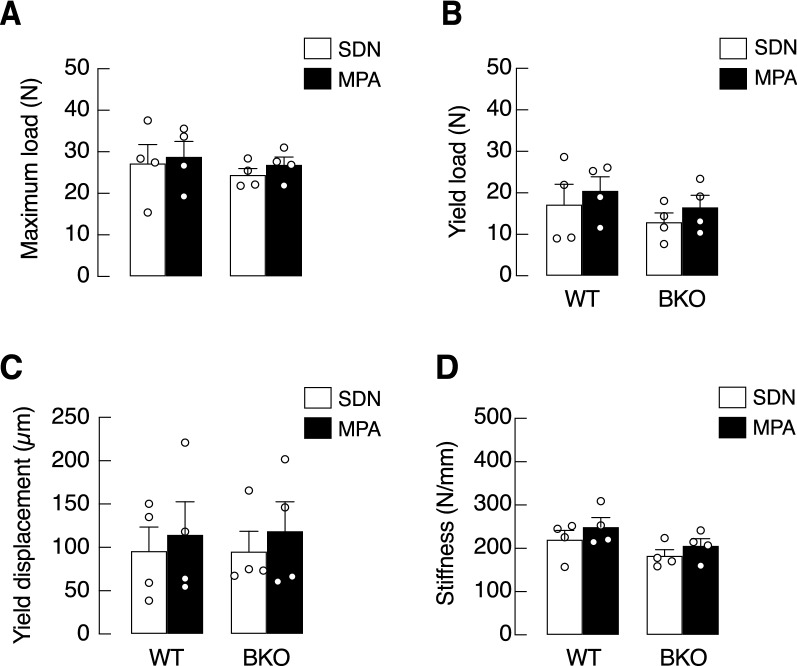
Mechanical properties of left femora of β-globin knockout (BKO) and wild-type (WT) mice after mild-intensity physical activity (MPA) training compared with the sedentary (SDN) control, i.e., (**A**) maximum load, (**B**) yield load, (**C**) yield displacement, and (**D**) stiffness. Values are means ± SE (n = 4).

**Figure 7:**
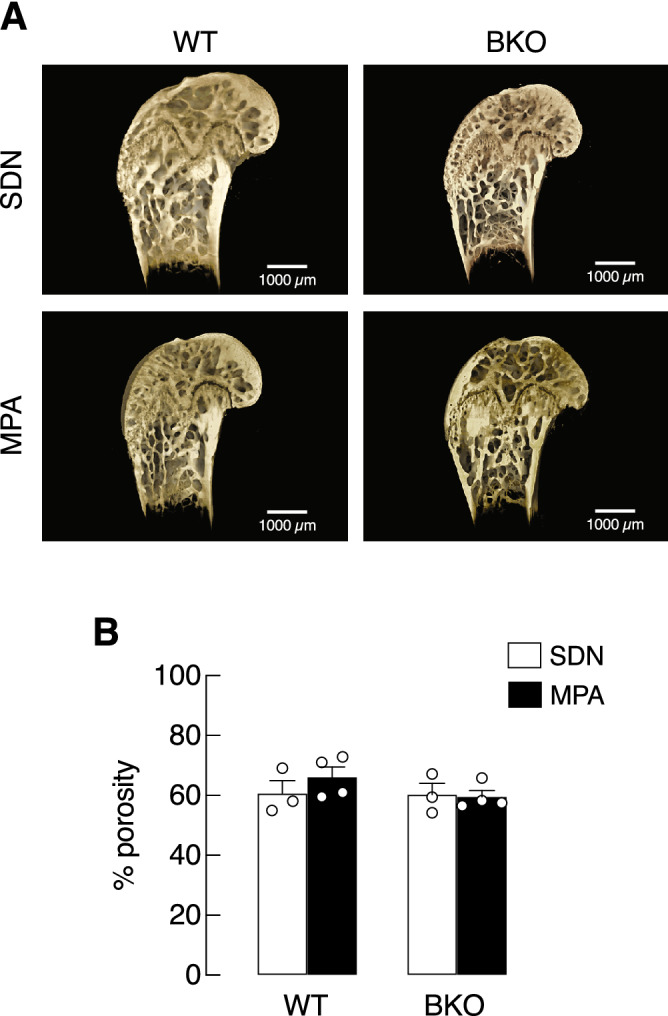
(**A**) A representative images of longitudinal microstructures of tibiae obtained from β-globin knockout (BKO) and wild-type (WT) mice after mild-intensity physical activity (MPA) training or sedentary (SDN) period as analyzed by SRXTM. (scale bar, 1000 µm). (**B**) Quantitative analyses of trabecular bone porosity in the proximal tibial metaphysis of BKO and WT mice subjected to MPA training or SDN control. Values are means ± SE (n = 3–4).

Regarding bone mechanical properties, BKO genotype and MPA did not affect maximum load, yield load, yield displacement, or stiffness (Fig. 6), suggesting that BKO mice was able to maintain bone strength despite exhibiting trabecular bone loss (Supplementary Fig. S1). Synchrotron radiation X-ray tomographic microscopy (SRXTM) confirmed the presence of trabecular thinning in the tibiae of BKO mice; however, the thinning appearance remained similar in BKO-SDN vs. BKO-MPA groups (Fig. 7A). Consistent with the absence of change in bone mechanical property, the percentage of porosity inside bone trabeculae and mineralized structure was not affected by BKO genotype or MPA, as determined by SRXTM (Fig. 7B).

## Discussion

Although exercise has been reported to generally improve bone health in thalassemia; however, some thalassemic patients have reduced capacity for endurance exercise^26,27^. Alternatively, MPA (e.g., walking or mild-intensity running) might be a more suitable choice of exercise to promote overall health. Here, we explored the effects of MPA on cardiac iron accumulation and calcium and bone metabolism in WT and BKO mice. We found that the body weights of WT and BKO mice were not affected by 3-month MPA, consistent with no change in the weights of internal organs and muscle (Fig. 1), and no noticeable changes in the serum levels of myokines, such as irisin and myostatin (Table 3), both of which are normally produced by rodent myocytes in higher amount during exercise^39^. The findings confirmed that the intensity of the present training protocol could be categorized as a mild physical activity and was suitable for the current thalassemic animal model with compromised cardiovascular reserve^2^.

Excess iron in thalassemia is usually deposited in various organs, including spleen, liver and heart, leading to enlargement of these organs (known collectively as hepatosplenomegaly). In general, hepatosplenomegaly seen in thalassemia is mainly caused by ineffective erythropoiesis that consequently leads to compensatory activation of an extramedullary erythropoiesis in other organs namely spleen and liver to produce red blood cells meet the body needs^40^. In contrast, cardiac hypertrophy in thalassemia is mainly a result of increased cardiac workload from body lack of oxygen and iron deposition, that exacerbate cardiac function leading to cardiomyopathy, a major cause of mortality in thalassemic patients^2^. Despite the cardiac iron content in BKO-SDN being higher than that of WT-SDN group, it was restored towards the WT level by MPA (Fig. 2C). This reduction of cardiac iron content was highly beneficial to thalassemic patients, as it improved cardiac function, reduced risk of cardiovascular diseases, and prolonged the patient’s life span^2,41,42^. Similarly, bone iron content was also completely restored to normal level by MPA (Fig. 2E). Therefore, despite being a mild intensity training, MPA successfully reduced iron accumulation in cardiac and bone tissues.

The exact underlying mechanism of MPA in restoring tissue iron content remains elusive. It is possible that MPA triggers cellular process that protects the cell from ROS-mediated cell injury, e.g., enhancing cellular oxygen consumption and mitochondrial ferritin (FtMt) function as seen after acute exhaustive exercise^43^. FtMt is a mitochondrial localized protein pertaining to ferrous oxidase activity, thus helping to reduce ROS production^43,44^. It has been reported that FtMt is presented in various tissues, e.g., heart, kidney, spermatocytes, and osteoblasts^43,45^. As mentioned earlier, FtMt was reported to prevent myocardial injury from oxidative stress induced by physical exercise^43,46^. Specifically, in normal rats, oxidative stress was generally increased with the intensity of physical activity, such as in vigorous or acute exhaustive exercise, and physical exercise at the same time increased the cardiac FtMt mRNA and protein expressions, perhaps a compensatory or protective mechanism. Physical exercise thus caused myocardial injury in FtMt null mice, which manifested high ROS production, apoptosis, severe cardiac mitochondrial injury and myocardial fibril disorganization^43^. It was, therefore, likely that minimal activity such as MPA was capable of reducing iron accumulation in the cardiac myocytes and bone tissue by enhancing FtMt function, thus protecting the tissues from oxidative stress.

Interestingly, MPA was also associated with an elevation of the circulating level of FGF-23, which normally functions as a calcium/phosphate-regulating hormone and a hypoferremic factor^9–11,35^. Besides being produced by bone, FGF-23 was also expressed in other tissues, e.g., intestine, spleen, kidney, liver, brain, and heart ^30,31,47^. We found that intact FGF-23 level in WT mice remained unchanged both in SDN and MPA groups throughout the 3-month study period. On the other hand, FGF-23 level in BKO-SDN rose in the second month and remain significantly higher than the WT level in the third month. Interestingly, the BKO-MPA group exhibited an earlier elevation in FGF-23 level at 1 month (Fig. 3A). A correlation study in BKO mice further revealed that the serum FGF-23 levels were inversely correlated with the cardiac iron content (Fig. 3B), thus agreeing with our hypothesis that the increased FGF-23 level might be a compensatory response to iron overload in thalassemia. The present finding was consistent with the previous reports of the elevated level of FGF-23 in thalassemia being associated with iron utilization^48,49^. For instance, Saki et al.^49^ demonstrated in β-thalassemia major patients a positive correlation between serum FGF-23 and ferritin from iron overload.

In another anemic model, i.e., chronic kidney disease (CKD), the reduction in renal function caused erythropoietin deficiency, low blood iron and inflammation^48^. In these CKD-induced anemic mice, a markedly elevated FGF-23 level could be attenuated by erythropoietin treatment, suggesting that iron utilization was one of the important drivers for FGF-23 production^48^. It is noteworthy that FGF-23 itself is probably a risk factor of cardiac hypertrophy in CKD with iron deficiency^50^, and perhaps whether FGF-23 becomes a protective or risk factor depends on the underlying body iron status of each individual. In addition, erythropoietin was probably elevated in tissue hypoxia associated with thalassemia^51^. It was evident that erythropoietin could increase circulating FGF-23 levels by upregulating the expression of FGF-23 transcript in hematopoietic cells^52,53^. Thus, although the sources of FGF-23 in BKO mice remain unclear, it can be released from multiple tissues, e.g., bone, intestine or hematopoietic cells, under modulation of many factors, e.g., erythropoietin and serum iron level.

Regarding the serum iFGF-23 vs. bone iron content, we postulated that the regulatory loop (or feedback mechanism) for FGF-23 release was first triggered by high serum iron (e.g., from erythrocyte breakdown and intestinal iron hyperabsorption), followed by bone iron accumulation. Once high FGF-23 level was maintained, bone iron accumulation was gradually alleviated as in Fig. 2E. As mentioned earlier, the exact cause of the elevated levels of circulating FGF-23 in thalassemia is still unclear. Another possible cause could be the consequence of thalassemia-induced hypoparathyroidism and low vitamin D production^1,7,8^. Since PTH normally stimulates the renal phosphate excretion and enhances bone resorption^54^, the low levels of PTH in thalassemia may lead to phosphate retention, which is known to be a strong stimulus for FGF-23 production. Furthermore, cardiac hypertrophy has been reported to be associated with the increased serum FGF-23^55^. Indeed, we observed significant increases in the circulating level of FGF-23 in both BKO-SDN and BKO-MPA groups. In addition, physical activity can increase circulating FGF-23 as seen in mice undergoing physical exercise training^56^. Li et al.^56^ demonstrated that all three levels of exercise intensities, i.e., acute exercise, exhaustive exercise and chronic exercise, could increase serum FGF-23. Furthermore, the exogenous FGF-23 treatment also extended the time to exhaustion and reduced exercise-induced ROS production^56^. Therefore, an early increase in FGF-23 level by MPA in our study possibly helped prevent excessive ROS production and enhance mitochondrial function.

It is well established that regular exercise and increased physical activity have benefit on bone health and intestinal calcium absorption^57,58^. Meanwhile, we have previously shown that the body uses FGF-23 to prevent excess intestinal calcium absorption through both local and systemic feedback loops^30,31,59,60^. In other words, FGF-23 is recognized as a negative regulator of intestinal calcium absorption^11^. For example, direct exposure of FGF-23 on duodenal tissue could completely abolish the 1,25(OH)_2_D_3_-induced calcium absorption^31^. Thus, BKO-MPA mice with elevated FGF-23 levels (Fig. 3A) was expected to respond differently from that of WT mice; otherwise a marked increase in FGF-23 would induce an unfavorable effect on the intestinal calcium absorption or calcium metabolism. Since MPA did not alter the duodenal calcium transport, epithelial electrical parameters (i.e., PD, *I*sc, TER) or calcium balance in BKO mice (Fig. 4B–G), MPA is considered a safe intervention that does not further compromise intestinal calcium absorption.

Regarding bone metabolism, physical exercise has many beneficial effects on the whole body and bone health. The low exercise capacity, poor physical fitness and fatigue in thalassemic patients thus contributed to poor bone health and decreased bone strength^16,27^. Thongchote et al.^16^ previously evaluated changes in bone microstructure in the 3-month-old BKO thalassemic mice, and found that forced endurance exercise could increase bone mass and also improved cortical and trabecular structure of bone. Static and dynamic bone histomorphometry revealed increased osteoblastic bone formation and reduced osteoclastic bone resorption^16^. Since forced endurance exercise with repeated exertion is not suitable for some thalassemic patients, MPA could be an alternative choice for reducing bone accumulation of toxic iron and normalizing calcium/phosphorus ratio, as demonstrated in the present study. Moreover, 3-month MPA significantly decreased the circulating levels of follistatin-like protein 1 (FSTL1) of BKO mice, which normally tended to have lower-than-normal FSTL1 levels (Table 3). Because of its roles as an antagonist of bone morphogenetic protein (BMP) signaling^61^, it is tempting to speculate that the MPA-induced reduction in FSTL1 level probably helps improve iron and bone metabolism, both of which are under complex regulation by various BMPs, e.g., BMP2, BMP4 and BMP6^61–63^. MPA also modulated the circulating levels of certain bone-derived humoral factors—osteocrin and osteonectin (Table 3)—although their pathophysiological importance in BKO mice requires further investigation.

It has been known that iron deposit in bone impairs osteoid maturation and profoundly compromises bone calcium and phosphate accretion, leading to anomalous calcium/phosphorus ratio, bone microcrystal defect and impairment of bone remodeling process^38^. In the present study, MPA was found to help preserve bone inorganic compositions by restoring calcium/phosphorus ratio. Specifically, calcium and phosphorus content analyses by FAAS revealed that 3-month MPA training was adequate for maintaining bone inorganic compositions in BKO mice (Fig. 5A–C). Although such a high calcium/phosphorus ratio in BKO-SDN was completely reverted to normal by MPA (Fig. 5D), MPA was not potent enough to affect bone mechanical properties (Fig. 6). Hence, evaluation of bone tissue by SRXTM showed that bone porosity of BKO-SDN and BKO-MPA mice were apparently similar, and change in trabecular microstructure was not observed (Fig. 7).

It is noteworthy that the present study has some limitations, such as the limited number of animals or small sample size. Since the BKO mice were very ill and the birth rate was somewhat below normal, the number of animals per group was minimized to make our experiments complied with the ARRIVE guideline. The absence of certain experiments pertaining to bone and calcium metabolism—such as mRNA expression levels of calcium transporters, Cyp27b1 and Cyp24a1, or related calcium transporters in the kidney, or trabecular bone histomorphometric analysis in MPA groups—was due to the fact that MPA did not alter intestinal calcium transport, calcium balance or bone porosity; therefore, we did not extend the research scope to cover those parameters.

In conclusions, despite being a mild-intensity intervention, MPA was able to produce beneficial effects on the heart and bone of BKO mice by reducing cardiac and bone deposition of toxic iron. This lower cardiac iron content was associated with elevated serum FGF-23, but BKO-MPA mice with high FGF-23 levels did not show a reduction in the duodenal calcium transport. Although regular exercise with higher intensity is often required to produce conspicuous changes in bone mechanical properties, the present positive outcome of MPA would be helpful for thalassemic patients with low level of physical fitness, and might contribute, in part, to be protective against thalassemic cardiomyopathy and osteopathy.

## Supplementary Information

Supplementary Figures.
