# Supplementary material for: Mild-intensity physical activity prevents cardiac and osseous iron deposition without affecting bone mechanical property or porosity in thalassemic mice
Source: Sci Rep. 2022 Apr 8;12:5959. doi: 10.1038/s41598-022-09997-x (PMC8993875; doi:10.1038/s41598-022-09997-x)
Supplement: Supplementary file 1 — Supplementary Figures. [file 41598_2022_9997_MOESM1_ESM.pdf]

Mild-intensity physical activity prevents cardiac and osseous iron deposition without affecting bone mechanical property or porosity in thalassemic mice

Narattaphol Charoenphandhu[1,2,3,4], Supagarn Sooksawanwit[1,2], Ratchaneevan Aeimlapa[1,2], Natchayaporn Thonapan[2,5], Pornpailin Upanan[6], Punyanuch Adulyaritthikul[1,2], Saowalak Krungchanuchat[1,2], Nattapon Panupinthu[1,2], Jarinthorn Teerapornpuntakit[2,7], Catleya Rojviriya[8], Kornkamon Lertsuwan[2,9], Saovaros Svasti[10], Kannikar Wongdee[2,6,*]

[1] Department of Physiology, Faculty of Science, Mahidol University, Bangkok 10400, Thailand
[2] Center of Calcium and Bone research (COCAB), Faculty of Science, Mahidol University, Bangkok 10400, Thailand
[3] Institute of Molecular Biosciences, Mahidol University, Nakhon Pathom 73170, Thailand
[4] The Academy of Science, The Royal Society of Thailand, Bangkok 10300, Thailand
[5] Molecular Medicine Graduate Program, Faculty of Science, Mahidol University, Bangkok 10400, Thailand
[6] Faculty of Allied Health Sciences, Burapha University, Chonburi 20131, Thailand
[7] Department of Physiology, Faculty of Medical Science, Naresuan University, Phitsanulok 65000, Thailand
[8] Synchrotron Light Research Institute (Public Organization), Nakhon Ratchasima 30000, Thailand
[9] Department of Biochemistry, Faculty of Science, Mahidol University, Bangkok 10400, Thailand
[10] Thalassemia Research Center, Institute of Molecular Biosciences, Mahidol University, Nakhon Pathom 73170, Thailand

Type of article: Research article

To whom correspondence should be addressed:
Kannikar Wongdee, Ph.D.
Faculty of Allied Health Sciences
Burapha University
Long-Hard Bangsaen Road
Chonburi 20131, Thailand
Tel : +66-3810-3618
E-mail: kannikar@go.buu.ac.th

Keywords: calcium balance study; cardiac iron accumulation; mild-intensity physical activity; synchrotron radiation X-ray microtomography; thalassemia

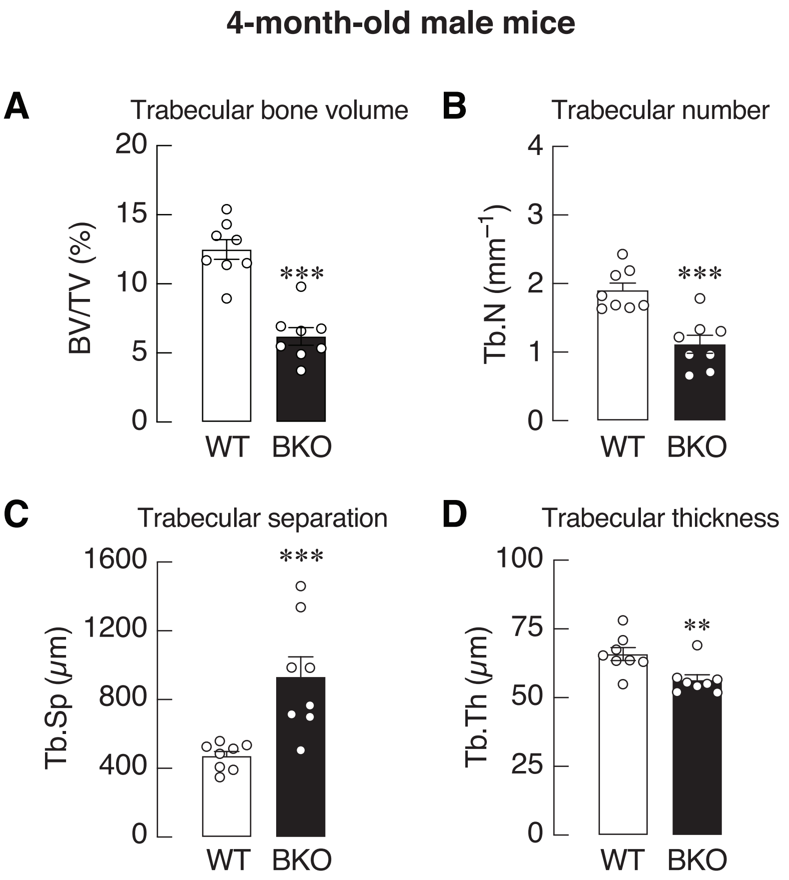

Figure S1: Microstructural analysis of proximal tibial metaphysis in 4-month-old β-globin knockout (BKO) and wild-type (WT) mice as determined by bone histomorphometry. (A) trabecular bone volume (bone volume fraction; BV/TV), (B) trabecular number (Tb.N), (C) trabecular separation (Tb.Sp), (D) trabecular thickness (Tb.Th). Values are means ± SE (n = 8). $^{**}P < 0.01$, and $^{***}P < 0.001$ vs. WT littermates.

4-month-old male mice

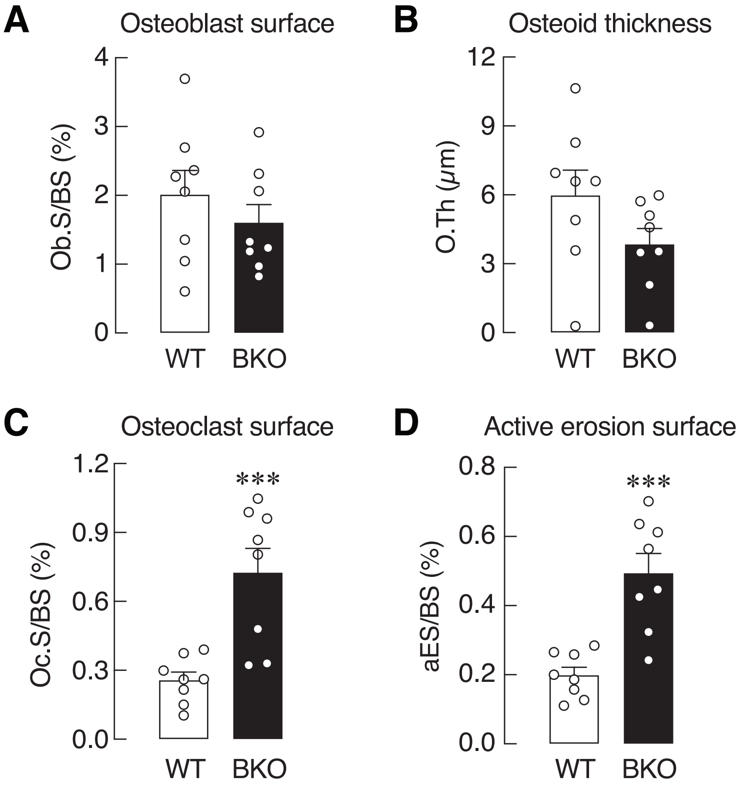

Figure S2: Microstructural analysis of proximal tibial metaphysis in 4 month-old β-globin knockout (BKO) and wild-type (WT) mice as determined by bone histomorphometry. Bone formation-related parameters: (A) osteoblast surface (Ob.S) normalized by bone surface (BS), (B) osteoid thickness (O.Th). Bone resorption-related parameters: (C) osteoclast surface (Oc.S) normalized by BS, and (D) active erosion surface (aES) normalized by BS. Values are means ± SE (n = 8). ***$P < 0.001$ vs. WT littermates.

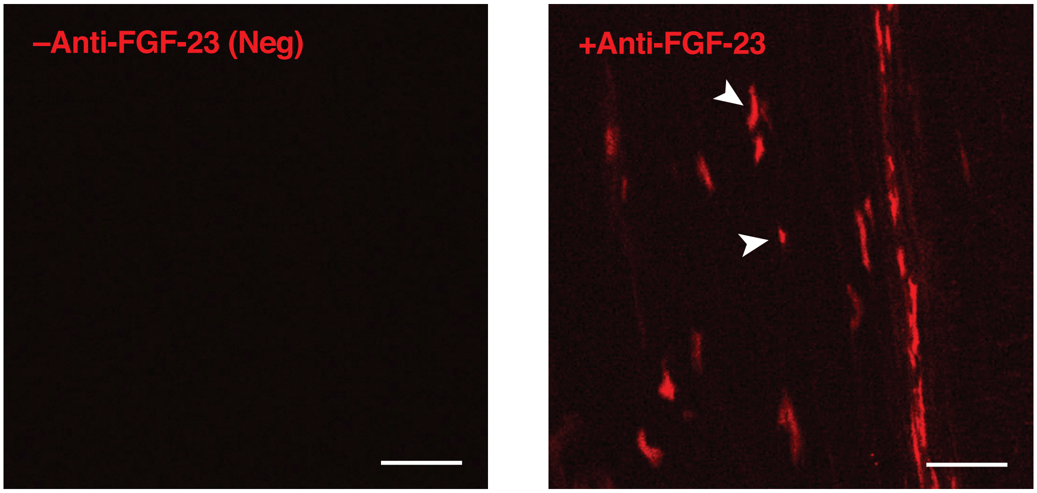

Figure S3: Representative photomicrographs showing the expression of FGF-23 proteins in the femoral cortical envelope of a 13-month-old normal rat. The fluorescent signals (red) are predominantly localized in osteocytes, which are known to abundantly express FGF-23. There is no positive signals in negative control (Neg). All images were captured by a SP8 multiphoton microscope. Arrow heads indicate osteocytes. Bars, 25 μM.
